# Toward Systematic Understanding of Flower Bud Induction in Apple: A Multi-Omics Approach

**DOI:** 10.3389/fpls.2021.604810

**Published:** 2021-03-25

**Authors:** Anton Milyaev, Julian Kofler, Iris Klaiber, Stefan Czemmel, Jens Pfannstiel, Henryk Flachowsky, Dario Stefanelli, Magda-Viola Hanke, Jens-Norbert Wünsche

**Affiliations:** ^1^Section of Crop Physiology of Specialty Crops (340f), Institute of Crop Science, University of Hohenheim, Stuttgart, Germany; ^2^Mass Spectrometry Unit, Core Facility Hohenheim (640), University of Hohenheim, Stuttgart, Germany; ^3^Quantitative Biology Center (QBiC) Tübingen, University of Tübingen, Tübingen, Germany; ^4^Institute for Breeding Research on Fruit Crops, Julius Kühn-Institut (JKI), Federal Research Centre for Cultivated Plants, Dresden, Germany; ^5^Agriculture Victoria, Department of Jobs, Precincts and Regions, Bundoora, VIC, Australia

**Keywords:** malus domestica, flower bud formation, RNA sequencing, proteomics, metabolomics, multi-omics

## Abstract

The induction of flower buds in apple (*Malus* × *domestica* Borkh.) is tightly connected to biennial bearing, which is characterized by alternating years with high (ON) and low or no (OFF) crop loads. In order to study this irregular cropping behavior, spur buds from ON- and OFF-trees of the biennial-bearing cultivar ‘Fuji’ and the regular bearing cultivar ‘Gala’ were collected. First, the time of flower bud initiation was precisely determined for both cultivars by histological analysis. Moreover, for a systematic understanding of flower bud induction in apple, the physiological and molecular mechanisms within the bud tissue were evaluated over four weeks prior to flower bud initiation by employing a multi-omics approach, including RNA sequencing, proteomic and metabolic profiling. Gene and protein enrichment analysis detected physiological pathways promoting and inhibiting early flower bud development. Metabolic profiles from the cropping treatments revealed a greater abundance of thiamine, chlorogenic acid, and an adenine derivative in spur buds from OFF-trees, whereas tryptophan was more abundant in the buds collected from ON-trees. Cultivar comparison indicated that chlorogenic acid was more abundant in ‘Gala’ than in ‘Fuji’ spur buds, whereas the opposite effect was found for tryptophan. Genes controlling tryptophan biosynthesis were not affected by ON- and OFF-treatments, but genes assigned to the metabolism of tryptophan into indoleacetate were differentially expressed between cultivars and treatments. The multi-omics approach permitted analyzing complex plant metabolic processes involved in early flower bud development and more specifically presumably in flower bud induction by tracing some pathways from gene to product level.

## Introduction

Flower induction, initiation, and differentiation are developmental stages that vegetative buds need to undergo on their way to become floral. Flower induction is commonly defined as a time point when a vegetative bud meristem perceives a signal to develop new tissue structures, so-called flower meristems. In contrast, flower initiation is characterized by distinct morphological, microscopically visible, meristematic changes in the bud (Foster et al., 2003; Hanke et al., 2007). While flower bud initiation can be determined by histological sectioning, the exact time of flower induction still remains obscure in many plant species such as apple (*Malus* × *domestica* Borkh.).

Based on mRNA expression data of flowering genes in apple shoot apices, it was assumed that the bud transition from induction to initiation could take around two weeks (Hanke et al., 2007). Early attempts to study flower initiation in apple were broadly reviewed by Jonkers (1979) and Monselise and Goldschmidt (1982). Merging the accumulated knowledge, some authors proposed hypothetical models of flower induction (Xing et al., 2015; Zuo et al., 2018); however, none of them could fully explain the genetic and physiological basis of this plant developmental stage. Understanding flower induction is of great importance for controlling crop load in perennial crops in order to establish regular annual cropping levels and to alleviate the production constraints associated with biennial bearing. The term biennial (or alternate) bearing in horticultural crops describes erratic yields when flowering density and in turn crop load in a given year strongly depends on the crop load of the previous year. It is frequently triggered by adverse environmental conditions, such as spring frost accompanied with flower damage, resulting in an OFF-year with low yield, followed by an ON-year with high yield of small-sized fruit before the repetitive cycle commences again with an OFF cropping status (Wünsche and Ferguson, 2005).

Initially, the reduced flower initiation rate on high-yielding apple trees was explained by sink–source interactions between fruit and buds concurrently developing within the growing season, with fruit being a stronger sink, thereby attracting proportionally more carbohydrate and in turn depriving the buds of the phloem-derived nutrients (Lenz, 1979). Numerous experiments indicated the importance of carbohydrate supply for flower bud development *in vitro*. Among the different sugars that have been tested in culture media, sucrose appeared to be the most effective to induce bud formation (Nitsch and Nitsch, 1967) and flower bud development (Jana and Shekhawat, 2011). Moreover, it was found that plant hormones and hormone-like acting compounds are strongly involved in the flower bud induction processes. In *Plumbago indica*, *in vitro* bud formation from callus was achieved in the presence of cytokinins and adenine and further promoted by adding indole-3-acetic acid (IAA). In the same study, flower bud formation was inhibited by application of three different gibberellins (Nitsch and Nitsch, 1967).

Recently developed omics analytical strategies have been driven largely by technological advances in mass spectrometry for proteomics and metabolomics and next-generation sequencing (NGS) assays, including RNA-Seq for transcriptomics. These high-throughput methods are cost-effective and target specific classes of biomolecules such as RNA transcripts, proteins, and primary or secondary metabolites. Despite the immense amount of data that can be obtained using each of those approaches, implementing only one of them, as for example done by Li et al. (2016, 2019) in apple and by Muñoz-Fambuena et al. (2013) in citrus, might not be sufficient to understand complex biological mechanisms such as flower induction in plants. With the appearance of gene-detection techniques, considerable research was devoted to the discovery of genes and transcription factors, which may promote or suppress flowering. Indeed, dozens of sequences were initially described as flowering regulators in Arabidopsis and later confirmed to be present as homologs in apple (Flachowsky et al., 2010, 2012; Haberman et al., 2017).

Despite these achievements in genomics, the current knowledge about the proteome and metabolome of apple is very limited and described so far only in the context of fruit development and maturation by Lin and Harnly (2013), Buts et al. (2016), and Li et al. (2016). Specifically, proteomic and metabolic data sets of apple buds in relation to biennial bearing are still missing. Many authors studied flower bud development by looking at the activity of particular genes (Zuo et al., 2018), transcription factors (Vimolmangkang et al., 2013), and proteins (Foster et al., 2007), which had already been discovered in other plants. To study the underlying biological processes involved in flower induction and to trace them from gene to product, it is necessary to combine several omics approaches in an attempt to better understand the interplay between genes, proteins, and metabolites determining the reproductive development of plants. The target and the novelty of the current work is the application of NGS in combination with two non-targeted omics approaches to develop a systematic understanding of the complex plant metabolic processes involved in flower bud induction in apple and tracing some pathways from gene to product level.

In the same experimental setup, we sampled apple buds for histological sectioning and revealed flower initiation time points for ‘Fuji’ and ‘Gala’ under field conditions in southwest Germany (Kofler et al., 2019). Considering these time points, we aimed to detect mobile signals potentially promoting or inhibiting flower bud induction such as peptides, phytohormones, phytohormone-like acting compounds, sugars, and secondary metabolites during 1–4 weeks prior to flower initiation and to link all the compounds of interest to genes and proteins involved in their biosynthesis and regulation. Specifically, the unknown mobile signals could influence or could be influenced by expression patterns of genes, determining the fate of the bud meristem. In order to test this hypothesis comprehensively on the transcript, protein, and metabolite level, we used a holistic multi-omics approach, targeting the flower induction mechanisms of the biennial-bearing apple cultivar ‘Fuji’ and of the regular bearing apple cultivar ‘Gala’. Although we acknowledge that there is a smooth transition from induction to initiation of flower buds, the signals in the gene to product pathway we found as early as four weeks prior to flower initiation can be assertively ascribed to flower induction.

Apple spur buds, which were used for multi-omics analyses, were collected from ON- and OFF-trees over four weeks leading up to flower initiation, covering the assumed period of flower induction. RNA extracted from the buds was analyzed using next-generation RNA sequencing; proteins and metabolites were detected using electrospray ionization (ESI) mass spectrometry in order to create multi-omics profiles of apple spur buds and to reveal the differences between ON- and OFF-trees. Here we summarize the results of RNA sequencing and non-targeted proteomic and metabolic profiling.

## Materials and Methods

### Plant Material and Experimental Design

**FIGURE 1:**
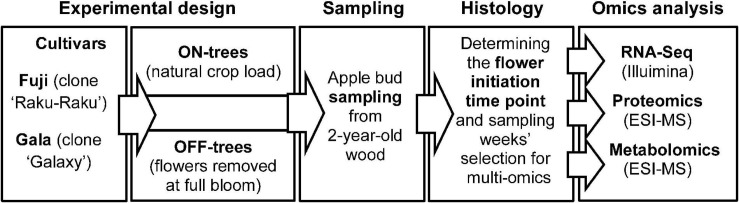
Schematic diagram of the plant material, treatments, sampling scheme, and analytical procedures used in this study.

The experimental apple orchard was located at the Centre of Competence for Fruit Cultivation near Ravensburg, Germany (47°46′2.89′′N 9°33′21.21′′E, altitude 490 m). The study was performed using 7-year-old ‘Fuji’ (clone “Raku-Raku”) and ‘Gala’ (clone “Galaxy”) apple trees (130 trees of each cultivar) grafted on M.9 rootstock. Trees of each cultivar were planted in two rows at 3 × 1 m spacing, respectively, trained as tall spindles of 3.5 m height and managed with standard irrigation and plant protection programs for the area. At the time of full bloom (30 April 2015), all flowers from randomly selected 65 trees per cultivar were removed by hand (OFF-trees), while the remaining trees were not thinned and maintained their natural flower density and hence high crop load (ON-trees). Subtending apple buds on 2-year-old spurs were collected weekly starting from four weeks after full bloom for 15 weeks until 2 September 2015. At each sampling week, 55 buds were collected from each of four randomly selected treatment-trees for proteomic and metabolic profiling and for RNA sequencing. After the brown bud scales were removed, the buds were placed into safe-lock tubes and snap-frozen in liquid nitrogen. The samples were stored at −80°C until used for analysis. The workflow including the plant material, treatments, sampling scheme, and analytical procedures used in this study is shown in Figure 1.

### Sampling Time Window for the Analyses

Observing flower bud meristem development by histological sectioning of apple buds sampled in the same experiment throughout the growing season of 2015, we identified flower initiation time points for ‘Fuji’ (75 days after full bloom, DAFB) and for ‘Gala’ (97 DAFB) (Kofler et al., 2019). The findings showed not only microscopically observed flower initiation time points but also suggested the predicted (modeled) time of the onset of flower initiation. The sampling window selection for the current study was based on the microscopically identified flower initiation time points. Assuming that a signal for flower bud induction must be detectable at least two weeks prior to flower initiation, we selected four sampling weeks prior to this event in order to perform omics analyses and to capture triggers of flower induction. Consequently, the multi-omics analysis covers sampling dates 48, 55, 63, and 68 DAFB, corresponding to 17 June, 24 June, 01 July, and 07 July for ‘Fuji’ and 68, 75, 83, and 89 DAFB, corresponding to 07, 14, 21, and 28 July for ‘Gala.’

### RNA Sequencing

Three trees, serving as replicates, were randomly selected for each of the two treatments (ON and OFF) at each of four sampling weeks chosen for the analysis. Five out of 55 collected buds per replicate were randomly taken for RNA extraction. This was performed using the InviTrap Spin Plant RNA Mini Kit (Invitek Molecular GmbH, Berlin, Germany) according to the standard protocol with the following modifications: Samples were first ground to powder using a cryogenic mixer mill (CryoMill, Retsch GmbH, Haan, Germany) cooled with liquid nitrogen. The grinding was done in safe-lock tubes with two steel balls (Ø = 5 mm) in one cycle of 10 s precooling and 6 min grinding at 25 Hz. A mixture of mercaptoethanol and lysis solution RP (1:100) was used for cell membrane disruption. Total RNA of each sample was eluted in 40 μl RNase-free water. The removal of contaminating DNA from the RNA preparations was achieved with the DNA-free DNA Removal Kit (Thermo Fisher Scientific, Waltham, United States). All the RNA samples were stored at −80°C until required.

RNA sequencing was conducted with c.ATG at the University of Tübingen, Germany. All samples were sequenced on a HiSeq 2500 (Illumina machine, Illumina Inc., San Diego, United States) using a paired-end (PE) mode and producing ∼10 million reads per sample. The resulting fastq files were demultiplexed and transferred to QBiC and analyzed on an HPC cluster of the University of Tübingen in a fully automated way using a Nextflow-based RNA-Seq pipeline^1^ (release 1.3). At the core of this workflow, *FASTQC* v0.11.8 (Andrews, 2010) was used to determine the quality of the FASTQ files. Subsequently, adapter trimming was conducted with *Trim Galore* v0.5.0 (Krueger et al., 2012). The *HISAT2* (v2.1.0) aligner was used to map the reads that passed the quality control against the GDDH13 apple genome version 1.1 from The National Institute of Agricultural Research (INRA)^2^ (Daccord et al., 2017). Annotation and fasta files were downloaded from there in January 2019. Read counting of the features (e.g., genes) was done with featureCounts v1.6.4 (Liao et al., 2014). For differential expression analysis, the raw-read-count table resulting from featureCounts was fed into the R package *limma* (v. 3.32.10) and *edgeR* (v. 3.18.1). First, the raw-read-count table was filtered for genes that had no expression in any of the samples. The remaining counts were then normalized by sequencing depth and log2 transformation using the edgeR functions calcNormFactors() and cpm() in order to meet the assumptions of linear models. To identify differentially expressed genes (DEGs) at each time point between ‘Fuji’ ON and ‘Fuji’ OFF as well as between ‘Gala’ ON and ‘Gala’ OFF conditions, a simple linear model was fitted to each gene consisting of a fixed effect for a combined factor of time (levels: 48, 55, 63, 68 DAFB for ‘Fuji’ and 68, 75, 83, 89 DAFB for ‘Gala’), genotype (levels: ‘Fuji’ and ‘Gala’), and treatment (levels: ON and OFF). *Limma* was then also used to extract pairwise contrasts including statistics for each gene including empirical Bayes moderated *p*-values which were finally adjusted for multiple testing by controlling the false discovery rate (FDR) using the Benjamini–Hochberg procedure (Benjamini and Hochberg, 1995). As threshold, a gene was called a DEG with a multiple adjusted *p* ≤ 0.05%. No log fold change filter criterion was applied for statistical assessment to find DEGs. For exploratory analysis, counts were normalized using the *DESeq2* package (v. 1.16.1) and visualized using standard packages in R (version 3.4.0). Translation of gene IDs into gene symbols was made using Blast2GO software (Conesa et al., 2005), where both apple genomes GDDH13 apple genome version 1.1 published by INRA and MalDomGD1.0 apple genome published by The National Center for Biotechnology Information were aligned. Gene symbols corresponding to gene IDs were chosen from the best-match column. For gene enrichment analysis and gene mapping to KEGG pathways, we used gene annotation software KOBAS 3.0 (Xie et al., 2011). All enriched pathways presented in the current work were selected from the main result output according to the *p* ≤ 0.05%. A Venn diagram was created using online software InteractiVenn (Heberle et al., 2015). Raw data can be accessed on ArrayExpress with the dataset identifier E-MTAB-9644^3^.

### Proteomic Profiling

Three out of the four trees per treatment and sampling week were randomly selected for proteomic profiling. Each replicate consisted of 16 buds, which were randomly taken from each 55-bud sample per tree. Sample preparation and analysis were performed according to Kofler et al. (2020). In order to compare ON- and OFF-treatments, relative quantification of proteins was performed.

Reverse hits, proteins identified only by site, and potential contaminants were removed from the dataset; LFQ intensities were log(2) transformed; and rows were filtered based on a minimum of three out of six potential valid values at each sampling date and a minimum of two unique peptides per protein. In order to perform a principal component analysis (PCA), missing values were imputed by random numbers from the normal distribution and a downshift of 1.8. For other statistical tests, missing values were not imputed; however, all the proteins found only in spur buds collected from ON- or only in the buds collected from OFF-trees were taken for enrichment analysis and KEGG search. A two-sided Student T-test at each date between the treatments was calculated with S0 = 0.1 and a permutation-based FDR of 0.05. Proteins which were statistically significant according to the T-test and exhibited a log2 fold change >0.5 were considered as differentially abundant. Differentially abundant proteins (DAPs) between treatments in at least one sampling week were considered for the subsequent KEGG pathway analysis. Raw data can be accessed on ProteomeXchange Consortium via the PRIDE repository with the dataset identifier PXD021716^4^.

### Metabolic Profiling

For metabolic profiling, one bud from each out of four trees per treatment at each of four sampling weeks was analyzed. Each bud was randomly taken from a mixture of 55 frozen buds collected from one tree representing one out of four replicates.

Metabolites from each bud were extracted in a separate safe-lock tube. Since the buds and safe-lock tubes had a slight weight deviation, all the empty tubes (64 in total) were numbered and weighed on the analytical balance to standardize the extraction conditions by adjusting the solvent volumes according to the net sample weight. Frozen buds were then quickly placed into corresponding tubes, weighed again in order to calculate net weights of the samples, opened to put in a precooled steel ball (Ø = 5 mm), and immediately returned to liquid nitrogen. The samples were ground to powder using a CryoMill (Retsch GmbH, Haan, Germany) at a frequency of 20 Hz with 10 s precooling and 4 min grinding. Each sample was eluted with ice-cold (−20°C) solution of 80% methanol and 20% distilled water, vortexed for 10 s, and placed on ice. The samples were kept frozen until cold 80% methanol was added. Methanol volumes were adjusted for each sample by using 120 μl methanol (80%) per 1 mg bud fresh weight. Steel balls were removed from the tubes using a magnet, and extracts were left at −20°C for 24 h for incubation. Incubated samples were centrifuged at 10,000 rcf for 4 min, and the supernatant containing metabolites was transferred into new tubes, which were kept at −20°C until required.

Non-targeted metabolic profiling was carried out at the Mass-Spectrometry Core Facility Unit at the University of Hohenheim, Stuttgart, Germany, using ultra-high-performance liquid chromatography (UHPLC) coupled with electro-spray ionization mass spectrometry (ESI-MS). The UHPLC unit Agilent 1290 Infinity LC System (Agilent Technologies, Inc., Santa Clara, United States) was equipped with an Acquity CSH C18 1.7 μm, 2.1 × 150 mm column (Waters Corporation, Milford, United States). Sample components were separated under the column temperature of 40°C using 0.1% formic acid (A) and acetonitrile with 0.1% formic acid (B) as solvents. From each extract, we used 7 μl for UHPLC injection with a flow rate of 400 μl min^–1^ and the following gradient: at 0 min 97% A and 3% B, from >0 to 15 min 80% A and 20% B, from >15 to 40 min 5% A and 95% B, and from >40 to 44 min 97% A and 3% B. Mass spectrometry was performed on a Thermo Fisher Scientific Q-Exactive Plus Orbitrap System (Thermo Fisher Scientific, Waltham, United States) in positive and negative ionization modes. The spray capillary voltage was set at 4.2 kV in positive mode and at 3.5 kV in negative mode, and desolvation temperature was 380°C. Mass spectra were acquired using a scan range from 140 to 1,500 m z^–1^ at a resolution of 70,000 full width half maximum (FWHM), an automatic gain control (AGC) target of 1.0 × 106, and a 100 ms maximum ion injection time. Data-dependent MS/MS spectra in a mass range of 200–2,000 m/z were generated for the five most abundant precursor ions with a resolution of 17,500 FWHM using an AGC target of 5.0 × 104 of and a 64 ms maximum ion injection time and a stepped collision energy of 15, 30, and 55 V. The measurement was started with a blank solution, after which the extracts (samples) were injected in a randomized order. Quality-control (QC) samples (a mixture of all the samples in equal proportions) were injected after every 10 measurements. Reference compounds were obtained from Merck KGaA (Darmstadt, Germany).

In order to compare ON- and OFF-treatments, relative quantification of metabolites was performed. For high-resolution accurate-mass data analysis and compound identification, we used Xcalibur 4.0.27.13 and Compound Discoverer 2.1.0.398 (Thermo Fisher Scientific, Waltham, United States) software. Fragmentation spectra and molecular masses of detected compounds were aligned to the references retrieved from the databases mzCloud, ChemSpider, Plant Metabolic Network (PMN), and PubChem. Additional statistical analyses of normalized peak areas were performed with Perseus 1.6.1.3 (Tyanova et al., 2016) and online software MetaboAnalyst 4.0 (Chong et al., 2019). PCA, including both cultivars, was not possible to perform due to analysis of samples from ‘Gala’ and ‘Fuji’ as two separate batches.

## Results

### Gene Expression and Proteomics

**FIGURE 2:**
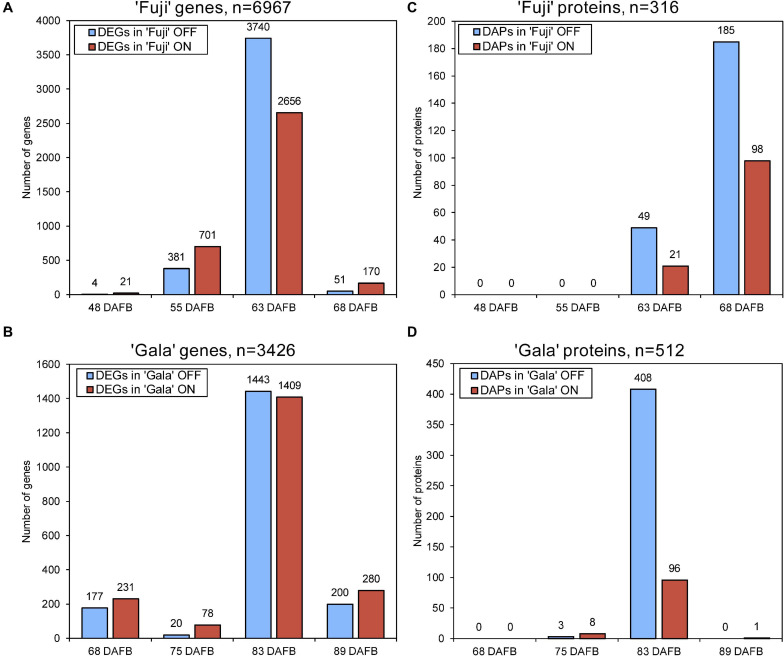
The number of differently expressed genes (DEGs) **(A,B)** and differentially abundant proteins (DAPs) **(C,D)** in ‘Fuji’ **(A,C)** and ‘Gala’ **(B,D)** between ON- and OFF-trees over 4 weeks prior to flower initiation. Flower initiation in ‘Fuji’ occurred 75 DAFB and that in ‘Gala’ 97 DAFB. The sums of the bar plots are not equal to the numbers (*n*) shown above each plot as n indicates the total number of unique DEGs and DAPs.

**FIGURE 3:**
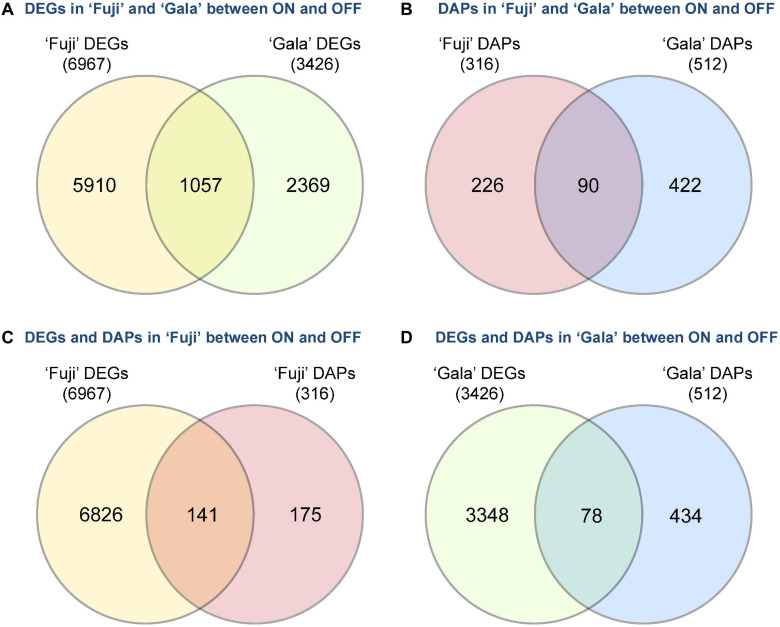
Crop load (ON, OFF)-induced number of differently expressed genes (DEGs) and differentially abundant proteins (DAPs) between **(A,B)** and within **(C,D)** the cultivars ‘Fuji’ and ‘Gala’ during 1–4 weeks prior to flower initiation.

Next-generation RNA sequencing of ‘Fuji’ and ‘Gala’ spur buds detected 40,916 genes (out of 46,558 total annotated genes) for which reads were successfully mapped to the double haploid ‘Golden Delicious’ GDDH13 genome version 1.1. In total, 6,967 genes in ‘Fuji’ and 3,426 genes in ‘Gala’ were differentially expressed between ON- and OFF-trees in any of the four selected sampling dates (Figures 2A,B). From those differentially expressed genes (DEGs), 1,057 were detected in both genotypes, while 5,910 DEGs were detected only in ‘Fuji’ and 2,369 DEGs only in ‘Gala’ (Figure 3A). The number of DEGs was not evenly distributed between the treatments and over the sampling dates. The majority of DEGs was observed 63 DAFB in ‘Fuji’ and 83 DAFB in ‘Gala’, corresponding to approximately 2 weeks prior to flower initiation in both cultivars (Figures 2A,B). At this developmental stage, a higher abundance of DEGs in spur buds from ‘Fuji’ OFF-trees was observed compared to ‘Fuji’ ON-trees, whereas the number of DEGs in ‘Gala’ was nearly the same in both treatments.

Proteomic profiling of ‘Fuji’ and ‘Gala’ spur buds resulted in a total number of 7,121 proteins detected by mass spectrometry. From them, 7,075 protein IDs could be linked to the corresponding genes identified in the transcriptomic analysis. Data quality filtering and statistical analyses of the proteomic profiles detected 316 DAPs in ‘Fuji’ and 512 DAPs in ‘Gala’ (Figures 2C,D). These DAPs were primarily found at 63 and 68 DAFB in ‘Fuji’, while in ‘Gala’ predominantly at 83 DAFB, a period, which corresponds to 1–2 weeks prior to flower initiation in both genotypes. From the total number of DAPs, 90 were common for both cultivars (Figure 3B). Comparison of transcriptomic and proteomic datasets showed that 141 DEGs in ‘Fuji’ and 78 DEGs in ‘Gala’ had corresponding DAPs (Figures 3C,D).

**FIGURE 4:**
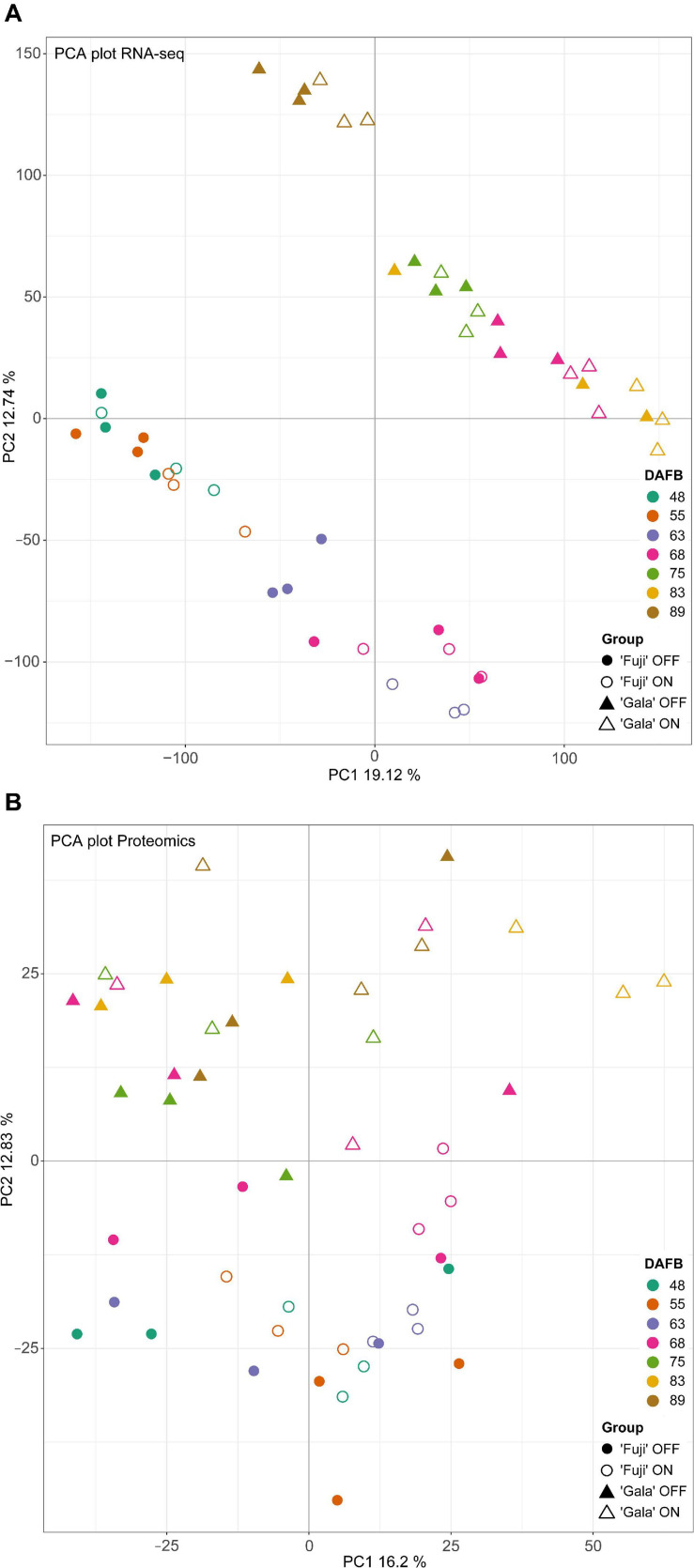
PCA analysis of transcriptomic **(A)** and proteomic **(B)** data.

Principal component analysis (PCA) of transcriptomic (Figure 4A) and proteomic (Figure 4B) data with variances of two main components of 31.86 and 29.03, respectively, showed clear distinctions between the studied cultivars. In the transcriptomic data, we observed prominent differences between ‘Fuji’ ON- and OFF-trees 63 DAFB, whereas in the proteomic data there was a clear distinction between ‘Gala’ ON- and OFF-trees 83 DAFB. Both time points correspond to approximately two weeks prior to flower initiation in both cultivars, respectively.

**FIGURE 5:**
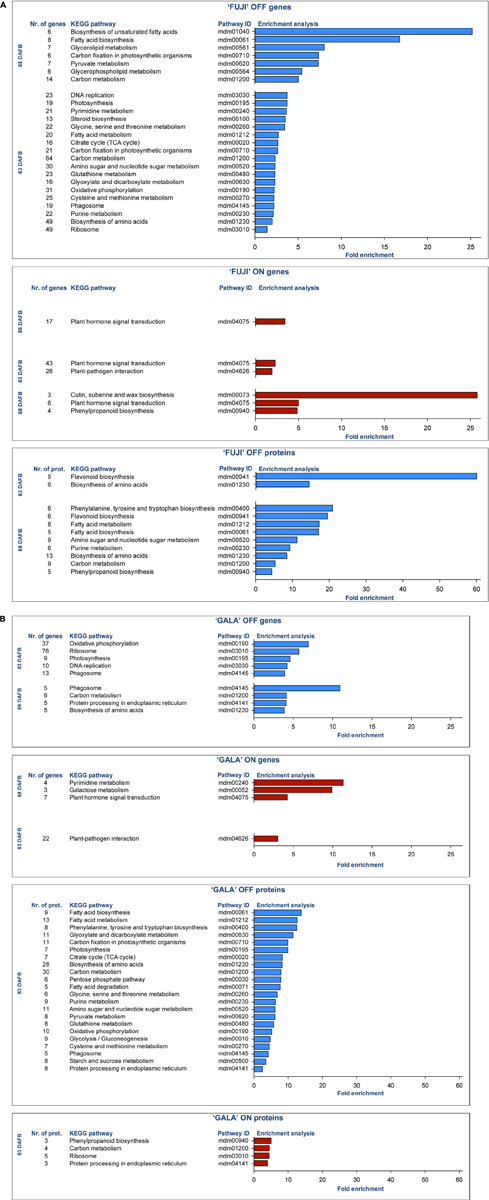
Enrichment analysis of KEGG pathways of differently expressed genes (DEGs) and differentially abundant proteins (DAPs) between ON- and OFF-trees of ‘Fuji’ **(A)** and ‘Gala’ **(B)**. Fold enrichment was calculated by comparing the background frequency of total genes or proteins annotated to that term in *Malus* × *domestica* to the sample frequency, representing the number of genes or proteins entered that fall under the same term (Dalmer and Clugston, 2019). In ‘Fuji’ ON, no significantly enriched pathways of DAPs were found. Detailed information about pathway entries and statistics is provided in Supplementary Tables 1–3.

DEGs and DAPs were mapped to KEGG pathways using gene list enrichment analysis with Kobas 3.0 (Xie et al., 2011). Enrichment analysis showed promoting (blue) and inhibiting (red) metabolic pathways for flower bud initiation in apple (Figures 5A,B), which were overrepresented in a given gene (or protein) list compared to the genome (or proteome) background information. Early flower bud development mechanisms in OFF-trees included metabolic pathways of carbon fixation, fatty acid biosynthesis, purine and pyrimidine metabolism, DNA replication, biosynthesis and metabolism of amino acids, steroid biosynthesis, amino sugar and nucleotide sugar metabolism, starch and sucrose metabolism, flavonoid biosynthesis, and others presented in Figure 5.

In spur buds from ON-trees of both cultivars, plant hormone signal transduction pathway, plant-pathogen interaction pathway, and phenylpropanoid biosynthesis were overrepresented (Figures 5A,B). The plant hormone signal transduction pathway in ‘Gala’ ON included 7 DEGs regulating signaling mechanisms of auxins, cytokinins (CKs), gibberellins (GAs), abscisic acid (ABA), and jasmonic acid (JA), whereas in the same pathway in ‘Fuji’ ON, 46 DEGs controlling signal transduction of at least eight known phytohormone groups—auxins, CKs, JA, GAs, ABA, salicylic acid (SA), brassinosteroids (BS), and ethylene—were detected (Supplementary Tables 1, 2). Enriched pathways of DEGs and DAPs within OFF-trees had partial overlaps. More distinct were enriched pathways of DEGs and DAPs in ON-trees. The cutin, suberin, and wax biosynthesis pathway was found to be enriched only in ‘Fuji’ ON and only in DEGs while there was no significantly enriched pathway based on DAPs in ‘Fuji’ ON-trees. In ‘Gala’, some metabolic pathways in ON-trees were detected, which were also found in OFF-trees of the same cultivar: carbon metabolism, ribosome and protein processing in endoplasmic reticulum, and pyrimidine metabolism.

### Metabolic Profiling

Besides transcriptomics and proteomics analyses, we also performed metabolic profiling of apple spur buds to detect the final products of gene expression activity and analyzed the abundances of small molecules, which may serve as mobile signals to trigger or inhibit flower induction. Computation of metabolic data revealed 1,491 mass/charge signals (features) in the positive ionization mode and 796 features in the negative ionization mode. From those, 1,140 unique features had MS^1^ isotope patterns and fragmentation spectra (MS^2^ spectra) of sufficient quality. Based on their sum formulas and tentative assignment in ChemSpider, PubChem, and Plant Metabolic Network (PMN), databases we were able to obtain general information about the compound classes that could be detected in apple bud tissue. These included amino acids and dipeptides, plant hormone-like acting substances, polyphenols and their glucosides, vitamins, triterpenoids, fatty acids, and unknown compounds, which are not yet included in the chemical databases. By the automated analysis of accurate m/z ratios and MS^1^-isotope patterns of the detected compounds, we obtained sum formulas for each of the metabolites. Based on the precise molecular mass search in the mentioned chemical databases and comparison of fragmentation spectra with existing reference spectra in mzCloud, 159 features could be linked to potential compounds (pre-identification step). After manual inspection of MS^1^ isotope patterns and fragmentation spectra in order to avoid false-positive identifications, the number of pre-identified compounds was further reduced to 111. They were characterized with robust MS^1^ signals, fragmentation spectra of sufficient quality, and measurable peak areas, which were used for further downstream processing.

**FIGURE 6:**
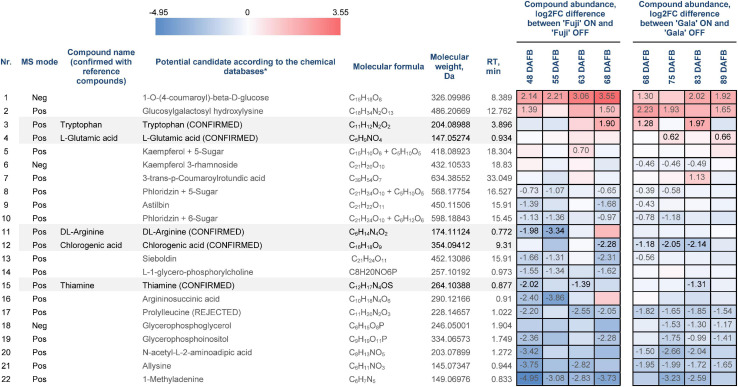
Differentially abundant metabolites (DAMs) in spur buds from ON- and OFF-trees of ‘Fuji’ and ‘Gala’. *Pre-identified metabolites according to mzCloud, ChemSpider, Plant Metabolic Network (PMN), and PubChem. “CONFIRMED” indicates identified organic molecules confirmed with reference compounds, “REJECTED” indicates structure and/or retention time is not confirmed using reference compounds. Negative log2FC values (blue) indicate a higher compound abundance in OFF, positive log2FC values (red) indicate a higher compound abundance in ON. Cells of the same color on the heat map indicate the same level of log2FC metabolite abundance difference between ON- and OFF-trees and do not indicate significant differences. Numbers on the heat map specify exact log2FC differences between ON- and OFF-trees and mark significant differences at multiple adjusted *p* ≤ 0.05.

Statistical data analysis of pre-identified 111 compounds revealed 22 compounds, whose abundances differed significantly between the spur buds from ON- and OFF-trees of either one or both cultivars in at least one out of four sampling weeks (Figure 6). The metabolite identification process is considered to be completed only if the structures of all candidate substances are confirmed by reference compounds. From those 22 compounds, 6 were available for purchasing as reference substances (prolylleucine, thiamine, chlorogenic acid, arginine, tryptophan, and glutamic acid). These compounds were used for verification of the database search result by comparing MS^1^ isotope patterns, retention times, and MS^2^ spectra to the corresponding features obtained by metabolic profiling. As a result, five reference compounds fully confirmed the proposed structures of thiamine, chlorogenic acid, arginine, tryptophan, and glutamic acid, whereas prolylleucine was not confirmed.

### Analysis of Candidates Across Omics Levels

**FIGURE 7:**
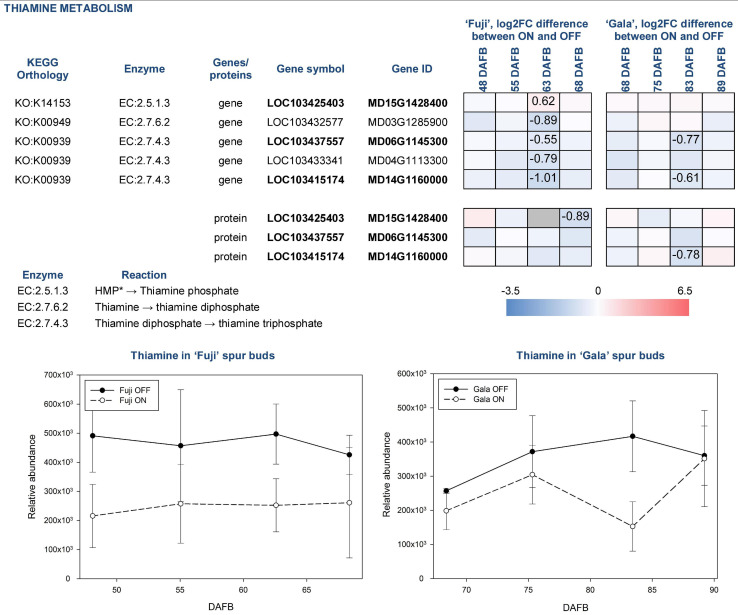
Relative abundance of thiamine in spur buds from ‘Fuji’ and ‘Gala’ with differently expressed genes (DEGs) between ON- and OFF-trees and corresponding proteins, which are assigned to the enzymatic reactions metabolizing the compound. *HMP—4-amino-5-hydroxymethyl-2-methylpyrimidine diphosphate. Information about the reactions and genes assigned to thiamine metabolism was provided from KEGG library. Bold font of gene and protein IDs indicates a link between a gene and a corresponding protein illustrated in the figure. Negative log2FC values (blue) indicate a higher gene expression or higher protein abundance in OFF, positive log2FC values (red)—in ON. Cells of the same color on the heat map indicate the same level of log2FC gene (protein) expression difference between ON- and OFF-trees and do not indicate significant differences. Numbers on the heat map specify exact log2FC differences between ON- and OFF-trees and mark significant differences at multiple adjusted *p* ≤ 0.05. Gray cells indicate no statistics possible due to “fill gap” function switched off (see Materials and Methods).

For analysis downstream of the individual omics analysis, we focus on the fully confirmed compounds, which were already described as compounds potentially influencing (inhibiting or promoting) early flower bud development, and also genes and proteins involved in their biosynthesis and metabolism. These compounds include thiamine, chlorogenic acid, and tryptophan. Thiamine was more abundant in spur buds from OFF-trees in both apple cultivars. The compound’s abundance in ‘Fuji’ ON and ‘Gala’ ON was at a similar level, whereas comparing spur buds from OFF-trees, it was slightly higher in ‘Fuji’. KEGG library indicates that thiamine originates from thiamine phosphate and can be further metabolized to thiamine diphosphate and thiamine triphosphate by thiamine diphosphokinase (EC:2.7.6.2; EC—enzyme commission number) and adenylate kinase (EC:2.7.4.3), respectively. The majority of DEGs and DAPs, which are assigned to those reactions, were also higher expressed in OFF-trees at 63 DAFB (‘Fuji’) 83 DAFB (‘Gala’) corresponding to 2 weeks prior to flower initiation in both cultivars (Figure 7).

**FIGURE 8:**
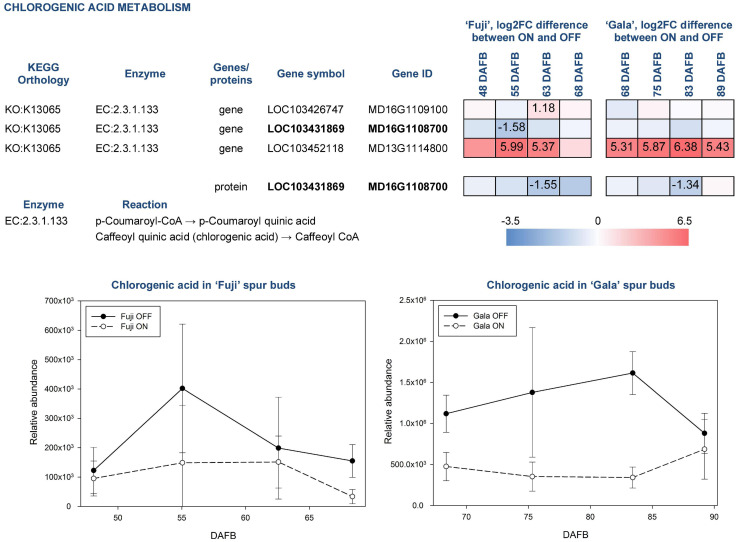
Relative abundance of chlorogenic acid in spur buds from ‘Fuji’ and ‘Gala’ with differently expressed genes (DEGs) between ON- and OFF-trees and corresponding proteins, which are assigned to the enzymatic reactions metabolizing the compound. For detailed figure explanation see Figure 7.

Chlorogenic acid is a product of coumaric acid metabolism, where enzymes belonging to shikimate O-hydroxycinnamoyltransferases (EC:2.3.1.133) catalyze multiple reactions. Among 24 genes assigned for these enzymatic reactions in KEGG, three DEGs and one DAP were identified in our study (Figure 8). According to a gene annotation in the apple genome, all of them were classified as HXXXD-type acyl-transferase family protein and showed distinct, sometimes even inverse, expression patterns. MD13G1114800 showed up to 6.38-fold greater expression in ON-trees of both cultivars. No proteins corresponding to this gene were detected. Another gene, MD16G1108700, was more highly expressed in spur buds from OFF-trees in both apple cultivars with a significant effect in ‘Fuji’ OFF at 55 DAFB. Its protein product was characterized by significantly higher abundance in OFF-trees in both cultivars two weeks prior to flower initiation. Average relative abundance of chlorogenic acid was 2.0-fold higher in ‘Fuji’ OFF-trees and 2.7-fold higher in ‘Gala’ OFF-trees compared to ON-trees with prominent cultivar differences. In ‘Gala’ ON and OFF, this compound was 4.3- and 5.6-fold more highly abundant than in ‘Fuji’ ON and OFF, respectively.

**FIGURE 9:**
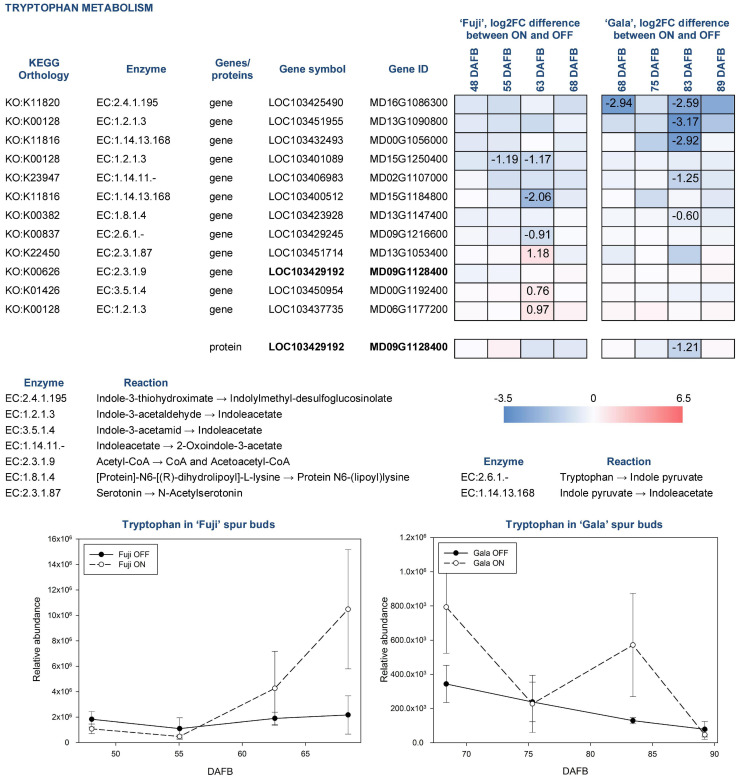
Relative abundance of tryptophan in spur buds from ‘Fuji’ and ‘Gala’ with differently expressed genes (DEGs) between ON- and OFF-trees and corresponding proteins, which are assigned to the indoleacetate biosynthesis. For detailed figure explanation see Figure 7.

Tryptophan in apple is synthesized by several reactions catalyzed by tryptophan synthase (EC:4.2.1.20), to which five genes were assigned using KEGG pathway analysis. However, none of them were differentially expressed between ON- and OFF-trees neither in ‘Fuji’ nor in ‘Gala’. Tryptophan detected in apple spur buds was characterized by 2.3-fold higher abundance in ‘Fuji’ ON compared to ‘Fuji’ OFF and by 2.1-fold higher abundance in ‘Gala’ ON compared to ‘Gala’ OFF. Cultivar comparison demonstrated that the relative abundance of tryptophan was much higher in ‘Fuji’ than in ‘Gala’ differing within both ON and OFF treatments, by about an order of magnitude (Figure 9). Furthermore, tryptophan was shown to increase in concentration toward flower initiation in ‘Fuji’ while it was more fluctuating in ‘Gala’.

To date, 66 genes are known to be involved in tryptophan metabolism in apple. Among them, 12 were found differentially expressed between ON- and OFF-trees in any cultivar, most of which were involved in indoleacetate biosynthesis. The expression patterns of those genes were not only cultivar specific but also treatment dependent. In particular, in ‘Gala’ OFF-trees, we detected DEGs for indoleacetate biosynthesis from indole-3-acetaldehyde (MD13G1090800) and indole pyruvate (MD00G1056000), whereas no genes showed significantly higher expression in ‘Gala’ ON-trees. In both ‘Fuji’ ON- and OFF-trees, we found DEGs coding aldehyde dehydrogenases (EC:1.2.1.3) converting indole-3-acetaldehyde into indoleacetate (MD15G1250400 in ‘Fuji’ OFF and MD06G1177200 in ‘Fuji’ ON). One gene coding indole-3-pyruvate monooxygenase (EC:1.14.13.168) that catalyzes reaction of indoleacetate biosynthesis from indole pyruvate (MD15G1184800) was more highly expressed in ‘Fuji’ OFF. Another gene of amidase enzyme (EC:3.5.1.4) leading to indoleacetate by catalyzing indole-3-acetamide was found more highly expressed in ‘Fuji’ ON (MD00G1192400). From the diversity of genes related to tryptophan metabolism, we could detect only one protein in the proteomic dataset (MD09G1128400). This protein was more highly abundant in ‘Gala’ OFF 83 DAFB.

## Discussion

Despite the fact that the apple genome has already been deciphered, even the newest high-quality *de novo* genome versions still cannot provide sufficient information about promotors and repressors of flowering and which specific metabolic pathways play a crucial regulatory role in early flower bud development. Many research groups attempted to identify genes that might induce flowering in higher plants; however, the majority of them used Arabidopsis as a model plant, whose ability to flower strongly depends on day length (Seo et al., 2011; Blümel et al., 2015). Nevertheless, there is evidence that some homologs of the flowering-regulating genes discovered in Arabidopsis also play a role in flower bud development in apple (Guitton et al., 2016). Transcriptomic analysis revealed numerous DEGs between ON- and OFF-trees two weeks prior to flower bud initiation and thereby showed that candidate genes for flower induction published in the literature is a small fraction of all transcripts differentially expressed between ON- and OFF-trees, which we could detect in this study. Gene and protein enrichment analyses illustrated metabolic pathway differences between spur buds collected from ON- and OFF-trees. In OFF-trees, we detected several enriched metabolic processes, which are assumed to lead to early flower bud development and meristem differentiation in apple. Among those were carbon fixation during cell photosynthetic activity and fatty acid biosynthesis, where enzymes from pyruvate and biotin metabolism pathways are involved (Rawsthorne, 2002). Fatty acids are then metabolized into different forms including glycerophospholipids, which are used to build cell membranes (Harwood, 2005). The metabolic profile of apple spur buds confirmed that fatty-acid-structured compounds were more abundant in OFF-trees (compounds 14, 18, and 19 in Figure 6). Besides that, in spur buds from OFF-trees we detected physiological pathways of amino acid biosynthesis and metabolism, metabolism of purine and pyrimidine, which serve as DNA and RNA constituents, DNA replication, starch and sucrose metabolism, and flavonoid biosynthesis.

Amino acid arginine, which was identified in the metabolic profiles of both apple cultivars, was significantly higher abundant in ‘Fuji’ OFF at 48 and 55 DAFB in comparison to ‘Fuji’ ON (Figure 6). Besides being a protein constituent, arginine serves as a nitrogen storage in plants and enables fine-tuning of the production of nitric oxide, polyamines, and potentially proline (Winter et al., 2015). Another detected amino acid, glutamic acid, showed higher abundance in ‘Gala’ ON at 75 and 89 DAFB. This compound is a precursor for the biosynthesis of arginine and proline, plays an essential role in nitrogen metabolism and can be synthesized by a number of physiological pathways (Forde and Lea, 2007). Moreover, glutamic acid was shown to be involved in plant stress responses and was associated with stress-triggered signal transduction (Qiu et al., 2020). However, there is so far no evidence on the involvement of arginine or glutamic acid in flower bud development. Amino acids phenylalanine, tyrosine, and tryptophan are immediate precursors for phenylpropanoid biosynthesis pathway that includes biosynthesis of flavonoids and chlorogenic acid in particular. Metabolic profiling of ‘Fuji’ and ‘Gala’ spur buds demonstrated that chlorogenic acid showed higher abundance in spur buds collected from OFF-trees. Cultivar comparisons within the same treatments showed several-fold higher abundance of chlorogenic acid in non-biennial cultivar ‘Gala’. In previous studies, chlorogenic acid was proven to inhibit IAA oxidase and therefore to protect auxin from its inactivation (Pilet, 1964). Furthermore, Lavee et al. (1986) reported that chlorogenic acid had auxin-like activity, affecting the growth of olive shoot apices cultivated *in vitro*. These findings suggest that chlorogenic acid might be involved in meristem development of a young bud. However, the mode of action of this compound in apple spur bud meristem remains unclear. The transcriptome and proteome of apple buds revealed the activity of enzymatic reaction EC:2.3.1.133 that metabolizes at least 3 derivatives of p-coumaric acid including p-coumaroyl-CoA and caffeoyl shikimic acid, which serve as chlorogenic acid precursors. The gene MD16G1108700 and the corresponding protein assigned to this reaction were detected in apple spur buds and were both more highly expressed in OFF-trees, whereas the MD13G1114800 gene showed significantly (up to 6.38-fold) higher expression in ON-trees with no detectable protein-product. Exploring the metabolic profile of ‘Fuji’ and ‘Gala’, we revealed that one compound from the metabolic dataset with *m* = 326.09986 and RT = 8.389 (compound 1 in Figure 6) showed similarity with a fragmentation spectrum of p-coumaric acid (peaks at 93.03, 119.05, and 163.04 m z^–1^), suggesting that this compound and chlorogenic acid, having p-coumaric acid as a structural base, may belong to the same metabolic pathway. The unknown compound showed up to 3.55-fold higher abundance in spur buds from ON-trees compared to OFF-trees; however, no direct connection of the biosynthesis of this compound with a high expression of the MD13G1114800 gene could be established. None of DEGs and DAPs related to the reaction EC:1.14.14.96, which converts p-coumaroylquinic acid (a product of EC:2.3.1.133) to chlorogenic acid, were found in our datasets.

Another compound that was more highly abundant in spur buds from OFF-trees of both cultivars was thiamine. This vitamin is frequently used in plant tissue culture and is proven to positively affect cell growth and development. It was reported that in *Plumbago indica*, *in vitro* bud formation from callus could be gained by adding the mixture of glycine, *myo*-inositol, nicotinic acid, thiamine, folic acid, and biotin routinely to culture media (Nitsch and Nitsch, 1967). In the multi-omics study, the thiamine biosynthesis pathway could also be detected at all three omics-levels of ‘Fuji’ and ‘Gala’ showing a higher abundance in spur buds from OFF-trees that supports previous findings. Though it was not possible to conclude whether thiamine directly contributes the flower induction by influencing the bud meristem formation, it is known that thiamine diphosphate plays a role as an enzymatic cofactor in universal metabolic pathways including glycolysis and the pentose phosphate pathway. Moreover, it has the same function in mitochondrial and chloroplastic pyruvate dehydrogenases. The latter provides acetyl-coenzyme A and NADH for biosynthesis of fatty acids (Goyer, 2010). The KEGG library indicates that in apple, thiamine is formed by cleaving of a phosphate group from thiamine phosphate, where enzymes classified as EC:3.1.3.100 are involved. Our analyses showed no DEGs or DAPs related to this enzyme group. The gene MD15G1428400 assigned to EC:2.5.1.3, thiamine phosphate synthase, was more highly expressed in ‘Fuji’ ON at 63 DAFB, whereas the other 4 genes assigned to EC:2.7.6.2 and EC:2.7.4.3 converting thiamine into thiamine diphosphate and latter into thiamine triphosphate, respectively, were more highly expressed in OFF-trees.

Among the pre-identified compounds with higher abundance in OFF-trees and thus potentially involved in early flower bud development, one compound showed a fragmentation spectrum similar to 1-methyladenine (peaks at 150.08, 133.05, 94.04, 82.04, and 55.03 m z^–1^, from which the last three are typical for adenine). It was characterized by up to 4.95-fold higher relative abundance in OFF-trees compared to ON-trees throughout all four sampling weeks in both apple cultivars. Notwithstanding all the efforts to classify the compound, we could not verify the proposed structure because of many differences in the fragmentation spectrum between the potential candidate and the reference spectrum of 1-methyladenine. It is well known that adenine serves as a base for the wide range of naturally occurring cytokinins (Kieber and Schaller, 2014). This fact raises an assumption that the unknown compound may represent a fragment, a precursor, or a product of metabolism of one of them.

The metabolic profile of ‘Fuji’ and ‘Gala’ spur buds suggests that the majority of pre-identified flavonoids were more abundant in OFF-trees (compounds 6, 8, 9, 10, 13 in Figure 6). This is in conformity with transcriptomic and proteomic profiles of spur buds collected from those cultivars, where the flavonoid biosynthesis pathway was significantly enriched in OFF-trees. The precise molecular masses and the fragmentation spectra of polyphenolic compounds indicated that they belong to the phloridzin and kaempferol aglycone type of flavonoids (compounds 5, 8, and 10 in Figure 6) with additional C5- and C6-sugar moieties attached to the aglycones. However, the precise structure of these phloridzin- and kaempferol-based flavonoids could not be determined. Scientific literature provided no evidence that flavonoids may have a direct influence on bud meristem development. However, the findings of Brown et al. (2001) and Peer et al. (2004) suggest that flavonoids play a role as negative auxin transport regulators, which would be in line with the higher abundance of flavonoids observed in OFF-trees.

In the metabolic dataset, tryptophan appeared in a cluster of small molecules, which were characterized by significantly higher abundance in spur buds from ON-trees. Moreover, cultivar comparison showed that tryptophan had 10-fold higher abundance in ‘Fuji’ spur buds compared to ‘Gala’. These differences make this compound and the metabolic pathways, in which tryptophan is involved, interesting for further studies. Since no genes assigned to tryptophan biosynthesis differed significantly in their expression between ON- and OFF-trees, it could be assumed that the accumulation of this compound in spur buds collected from ON-trees may be the result of suspended tryptophan conversion to other primary or secondary metabolites. Transcriptomic analysis showed that the majority of DEGs from the tryptophan metabolism pathway were assigned to indoleacetate biosynthesis, an active auxin form in plants. However, none of the known auxin forms could be detected in apple buds by the applied analytical method.

Transcriptomic and proteomic analyses indicated an involvement of plant hormone signal transduction pathways in bud meristem fate. Based on this information, in our metabolic approach, we used reversed-phase (RP) chromatography which is known to be suitable to analyze hydrophobic compounds such as phytohormones (Pan et al., 2010). However, none of the known plant hormones could be successfully detected using the non-targeted metabolomics, most likely due to the low abundance of these compounds in apple bud tissue compared to other metabolites. Therefore, a further step toward a systematic understanding of flower induction in apple would be the determination of plant hormone profiles by targeted mass spectrometry analyses using extraction protocols specifically designed for the analyses of phytohormones (Farrow and Emery, 2012; Urbanová et al., 2013).

NGS and proteome analysis revealed several sugar metabolism and interconversion pathways that are actively ongoing in spur buds, which develop flower meristem, indicating that particular sugar forms might play an important role in flower bud induction and initiation. However, the majority of MS signals obtained from different sugar forms found in metabolic profiles of apple spur buds could not be interpreted because the clear separation of sugar molecules with the same molecular mass using reversed-phase chromatography could not be achieved. In order to enlarge the knowledge about the involvement of simple sugar forms, such as mono-, di-, and trisaccharides in flower induction in apple, sugar analysis in apple buds is also essential. Once performed, it would help to link the activity of genes and corresponding proteins to specific sugar compounds.

The picture of flower bud induction mechanisms in apple is far from complete. In the recent years, the understanding of plant flower organ formation mechanisms has strongly advanced; however, it is still unclear what the initial trigger for floral meristem formation is and how the fruit may inhibit flower development in the adjacent spur buds. The second question may be answered by determining which metabolites that showed higher abundance in spur buds from ON-trees are originated from the fruit. Once determined, it would narrow down the search of candidate genes, which are affected by the unknown mobile signal. In the apple metabolome, from 1,140 features with robust signals detected by non-targeted metabolomics, only 111 could be annotated with any potential compound from chemical databases, indicating that only 10% of compounds found in apple spur buds could be claimed as “knowns.” Besides the 22 compounds presented in the current work, nearly 70 hitherto unknown metabolites were found to be differentially abundant between spur buds from ON- and OFF-trees on a time-series scale.

In summary, the multi-omics approach applied for the identification of flower induction signaling molecules in apple allowed observing complex plant metabolic processes and tracing some pathways from gene to product level. The data suggests that thiamine, chlorogenic acid, and an adenine derivative play a role in metabolic pathways promoting early flower bud development in apple. Tryptophan was found to be more abundant in spur buds collected from high-cropping (ON) trees compared to non-cropping OFF-trees. Cultivar comparison (biennial cultivar ‘Fuji’ vs. non-biennial cultivar ‘Gala’) revealed 4.3–5.6-fold higher abundance of chlorogenic acid in ‘Gala’ spur buds, whereas tryptophan was 10-fold higher abundant in spur buds collected from ‘Fuji’. Genes controlling tryptophan biosynthesis were not affected by ON- and OFF-treatments; however, genes regulating tryptophan metabolism to indoleacetate showed significant expression differences between treatments and cultivars. At transcriptomic and proteomic levels, in apple spur buds collected from OFF-trees, metabolic pathways associated with tissue growth and development were detected that potentially result in a promoting effect on early flower bud development. In contrast, in spur buds from ON-trees, the plant hormone signal transduction pathway was enriched, suggesting the involvement of hormonal metabolites in determining the fate of the apple bud meristem.

## Data Availability Statement

The proteomic data are available on Proteomics Identifications Database (PRIDE) under the ID PXD021716. The RNAseq data are accessible on ArrayExpress under the ID E-MTAB-9644.

## Author Contributions

AM and JK carried out the experiment. AM performed the proteomics and metabolomics data analysis with support of SC, IK, JP, JK, and DS and wrote the manuscript with support of JW, HF, and SC. IK and JP conducted MS analysis of proteins and metabolites. SC performed RNA sequencing and RNA-Seq downstream analysis. JW, HF, and MH supervised the project. All authors provided critical feedback and helped to shape the research, analyses, and the manuscript.

## Conflict of Interest

The authors declare that the research was conducted in the absence of any commercial or financial relationships that could be construed as a potential conflict of interest.

## Footnotes

1 https://github.com/nf-core/rnaseq

2 https://iris.angers.inra.fr/gddh13/

3 https://www.ebi.ac.uk/arrayexpress/experiments/E-MTAB-9644/

4 https://www.ebi.ac.uk/pride/

## Supplementary Material

Supplementary Table 1 Fuji gene enrichment analysis.

Supplementary Table 2 Gala gene enrichment analysis.

Supplementary Table 3 Fuji and Gala protein enrichment analysis.

## Abbreviations

ABA: abscisic acid
BS: brassinosteroids
CK: cytokinins
DAFB: days after full bloom
DAP: differentially abundant protein
DAM: differentially abundant metabolite
DEG: differentially expressed gene
GA: gibberellins
JA: jasmonic acid
KEGG: Kyoto Encyclopedia of Genes and Genomes
log2FC: log2 fold change
MS: mass spectrometry
NGS: next-generation sequencing
IAA: indole-3-acetic acid
RT: retention time
SA: salicylic acid.
